# Impact of novel SNPs identified in *Cynara cardunculus* genes on functionality of proteins regulating phenylpropanoid pathway and their association with biological activities

**DOI:** 10.1186/s12864-017-3534-8

**Published:** 2017-02-17

**Authors:** Ana Margarida Ferro, Patrícia Ramos, Olinda Guerreiro, Eliana Jerónimo, Inês Pires, Carmen Capel, Juan Capel, Rafael Lozano, Maria F. Duarte, M. Margarida Oliveira, Sónia Gonçalves

**Affiliations:** 1 Centro de Biotecnologia Agrícola e Agro-Alimentar do Alentejo (CEBAL), Instituto Politécnico de Beja (IPBeja), Rua Pedro Soares, 7801-908 Beja, Portugal; 2 Instituto de Tecnologia Química e Biológica António Xavier, Universidade Nova de Lisboa (ITQB NOVA), Av. da República, 2781-901 Oeiras, Portugal; 3 Centre for Research in Ceramics and Composite Materials (CICECO) and Department of Chemistry, University of Aveiro, 3810-193 Aveiro, Portugal; 4 Centro de Investigação Interdisciplinar em Sanidade Animal (CIISA), Faculdade de Medicina Veterinária, University of Lisbon, Avenida da Universidade Técnica, 1300-477 Lisboa, Portugal; 5 Centro de Investigación en Biotecnología Agroalimentaria (BITAL), Universidad de Almería, 04120 Almeria, Spain; 6 Wellcome Trust Sanger Institute, Wellcome Trust Genome Campus, Hinxton, CB101SA Cambridge, UK

**Keywords:** *Cynara cardunculus*, Chlorogenic acid, C3′H, HQT, High-resolution melting, SNPs, Haplotype, Antioxidant activity, Antithrombin activity, Association analysis

## Abstract

**Background:**

*Cynara cardunculus* L. offers a natural source of phenolic compounds with the predominant molecule being chlorogenic acid. Chlorogenic acid is gaining interest due to its involvement in various biological properties such as, antibacterial, antifungal, antioxidant, hepatoprotective, and anticarcinogenic activities.

**Results:**

In this work we screened a *Cynara cardunculus* collection for new allelic variants in key genes involved in the chlorogenic acid biosynthesis pathway. The target genes encode *p*-coumaroyl ester 3′-hydroxylase (C3′H) and hydroxycinnamoyl-CoA: quinate hydroxycinnamoyl transferase (HQT), both participating in the synthesis of chlorogenic acid. Using high-resolution melting, the C3′H gene proved to be highly conserved with only 4 haplotypes while, for HQT, 17 haplotypes were identified *de novo*. The putative influence of the identified polymorphisms in C3′H and HQT proteins was further evaluated using bioinformatics tools. We could identify some polymorphisms that may lead to protein conformational changes. Chlorogenic acid content, antioxidant and antithrombin activities were also evaluated in *Cc* leaf extracts and an association analysis was performed to assess a putative correlation between these traits and the identified polymorphisms.

**Conclusion:**

In this work we identified allelic variants with putative impact on C3′H and HQT proteins which are significantly associated with chlorogenic acid content and antioxidant activity. Further study of these alleles should be explored to assess putative relevance as genetic markers correlating with *Cynara cardunculus* biological properties with further confirmation by functional analysis.

**Electronic supplementary material:**

The online version of this article (doi:10.1186/s12864-017-3534-8) contains supplementary material, which is available to authorized users.

## Background

Plants have always been considered an important source of biologically active compounds, mostly associated with health improvement. In the last decade nutritional therapy and phytotherapy appeared as new concepts related to the prevention and treatment of different human diseases [92]. In this context, the extraction and identification of plant-derived bioactive compounds has become an important target in agriculture and food science research. Although *Cynara cardunculus* L. (*Cc*) has been known for its therapeutic activities and used in folk medicine since ancient times, renewed interest has arisen through recent studies on its chemical and biological characterization [23, 72, 73].

*Cynara cardunculus* is native to the Mediterranean region and well adapted to hot and dry climates [33]. It comprises three botanical varieties: the globe artichoke (var. *scolymus* L.); the cultivated cardoon (var. *altilis* DC.); and the wild cardoon (var. *sylvestris* (Lamk) Fiori) [70], which is considered to be the wild progenitor of the first two varieties [2, 45, 74]. Globe artichoke is economically important mainly in Italy, Spain, France and Turkey, having been selected for production of edible immature capitula (“heads”) used in traditional cuisine [37]. Cultivated *Cc* has different potential applications, namely the production of solid biofuel [25, 31, 69], seed oil [18, 52], biodiesel [21, 22], paper pulp [1, 33] and green forage [24]. In Portugal, cultivated and wild cardoon are the most used varieties. Cardoon has been selected for its fleshy stems and leaf petioles, which are consumed as vegetables, while flowers are used as a source of aspartic proteases for milk clotting during cheese production [25]. However, *Cc* also has potential for industrial use as a source of bioactive phenolic compounds [72]. In artichoke, leaf extracts have been reported as having relevant biological properties, such as antibacterial [93], antifungal [94], antioxidant [42, 72, 88], hepatoprotective [3], anticarcinogenic [57, 58, 65] and inhibition of cholesterol biosynthesis [5, 30, 43]. In previous experiments we found that cardoon phenolic extracts prepared from different organs showed a high antioxidant and reduction capacity as well as a strong antitumorigenic effect in a human breast cancer cell line [86]. Many of the bioactivities beneficial for human health have been attributed to the presence of phenols, particularly caffeic acid derivatives such as 5-caffeoylquinic acid (chlorogenic acid, CGA) and di-caffeoylquinic acids and flavonoids [3, 48, 58, 72, 86, 88, 93], These findings have encouraged the use of breeding programs aimed at increasing their levels in crops.

The chlorogenic acid biosynthesis pathway has been studied in different *Solanaceae* species, such as tomato [60], tobacco [36, 56], eggplant [34] as well as in other families including apple [51], coffee [44], and artichoke [16, 17, 61, 62, 78]. The formation of CGA is still not well defined but two possible pathways were suggested: (1) hydroxylation of *p-*coumaroylquinic acid to CGA via *p*-coumaroyl ester 3′-hydroxylase (C3′H); and (2) hydroxylation of *p-*coumaroylshikimic acid to caffeoylshikimic acid via C3′H, followed by conversion to caffeoyl-CoA, a substrate of hydroxycinnamoyl-CoA: shikimate hydroxycinnamoyl transferase HCT. Caffeoyl-CoA is then converted to CGA by trans-esterification with quinic acid via hydroxycinnamoyl-CoA: quinate hydroxycinnamoyl transferase (HQT) activity [17]. In globe artichoke, the mRNAs coding for two hydroxycinamoyltransferases (HCT and HQT) and for the *p*-coumaroyl ester 3′-hydroxylase (C3′H) involved in chlorogenic acid synthesis, have been identified [16, 17, 61, 78]. Several studies have previously shown the role of *C3′H* and *HQT* genes in the accumulation of phenylpropanoid pathway metabolites. In tomato, *HQT* overexpression leads to CGA accumulation, improving plant antioxidant capacity [66].

Artichoke and cardoon have been domesticated since Roman times, and artichoke breeding programs were established since the XX century for quality and crop productivity improvement [19, 26, 50, 59, 79, 84]. However, despite the economic and pharmacological value of *C. cardunculus* its improvement through breeding is still limited. An increased knowledge of genes involved in the biosynthesis of selected compounds could help in precision breeding focused on enriching their levels.

Therefore, the objective of this work was to screen a natural population of *C. cardunculus* plants for single nucleotide mutations in selected genes of the phenylpropanoid pathway to characterize their genetic variability. Sequences of *C3′H* and *HQT* genes were selected and the high-resolution melting technique (HRM) was applied to identify single nucleotide polymorphisms (SNPs). Moreover, the CGA content, the antioxidant and the antithrombin activities were also evaluated in an attempt to correlate chemical and biological variations with the different *Cc* haplotypes identified.

## Methods

### Plant materials

A collection of 29 accessions comprising 127 individuals of *Cynara cardunculus* from different sources was established at CEBAL. This material included 25 accessions of wild cardoon, 3 of cultivated cardoon and 1 of artichoke. The material was obtained from Portugal (2 accessions), Spain (4 accessions), Italy (18 accessions from 4 regions) and England, Moldavia, Hungary, Algeria and Norway with 1 accession each (see Additional file 1 for details). Seeds were washed in distilled water and 7–8 seeds were placed on wetted paper in Petri dishes. The plates were capped and stored at 4 °C for 4 days to break seed dormancy. The plates with seeds were then transferred to a growth chamber with a 16/8 h photoperiod, temperature of 22 °C and 70% humidity to germinate. Five days after germination the seedlings were transplanted to pots with soil (90%) and sand (10%) and maintained at room temperature with regularly watering. After 90 days, young leaves were collected and immediately used for DNA isolation.

Four months after germination, plants were transplanted to larger pots with the same proportions of soil and sand and transplanted to the field. For the phenotypic traits analysis 1 individual each from Portugal, Napoli, Hungary, Norway, Moldavia and England, 3 individuals from South Italy and Rome, 2 individuals from North Italy and 6 from Spain were selected as representative of the *Cc* haplotypes diversity comprising 20 individuals (1, 3 and 16 individuals, respectively, of artichoke, cultivated and wild variety). Nine-month-old leaves were collected and preserved at −80 °C until phenotypic analysis.

### High-resolution melting (HRM) analysis

DNA was extracted from 100 mg of young leaf tissue using the DNeasy Plant Mini kit (Qiagen, Germany) and quantified on a 1% agarose gel. High-resolution melting-specific primers for *C3′H* and *HQT* coding sequences (CDS) [GenBank: FJ225121.1 and DQ915590.1, respectively] were designed in order to amplify overlapping segments of DNA coding sequences using Primer 3 software (http://bioinfo.ut.ee/primer3-0.4.0/) (Additional files 2 and 3). The presence of *C3′H* and *HQT* gene polymorphisms associated with natural variation was assessed by HRM, performed as described by Han et al. [35]. PCR amplifications were performed in 96-well plates, in a total volume of 10 μL containing 0.5 ng of genomic DNA, 1 μM of forward and reverse primer, 1× HotShot Diamond™ PCR Mastermix (Clent Life Science, UK) and 1 μL of LCGreen® Plus + Melting Dye (Idaho Technologies, Salt Lake, UT, USA). Amplification reaction was performed in a thermocycler Mastercycler® pro Eppendorf (Hamburg, Germany) as follows: 94 °C, 10 min; 45 cycles of 94 °C, 30 s, 62 °C, 30 s and a final cycle at 94 °C, 30 s and 26 °C, 30 s. Melting profiles were analysed in a LightScanner® (Idaho Technology, Salt Lake, UT, USA) by increasing the temperature by 0.5 °C s^−1^ from 65 to 95 °C. Melting data were analysed using the LightScanner Software 2.0 (Idaho Technologies). After data calibration and normalization, samples were grouped based on the shape of the normalized melting curves (profile) and the sensitivity level was adjusted to distinguish all genotype groups.

### Haplotype analysis and sequence validation

From each HRM profile, three DNA samples were amplified twice and sequenced to confirm the results obtained. PCR amplifications products were purified and sequenced by Beckman Coulter Genomics (Takeley, UK), with an ABI 3730xl sequencing platform. Sequences obtained were assembled and single nucleotide polymorphisms (SNPs) were identified in the different individuals. SNPs analyses that resulted in aminoacid (a.a) change allowed the identification of specific haplotypes. Based on previous background information of cultivated cardoon from Beja, Portugal, one individual (AC2) of this accession was chosen as reference.

Haplotype analysis was performed using SNiPlay Pipeline (http://sniplay.cirad.fr/cgi-bin/home.cgi) [20]. A network analysis of the different haplogroups was performed using Haplophyle software with default parameters (http://haplophyle.cirad.fr/Haplophyle/).

### Prediction of protein structural changes

ExPAZy translate tool (http://web.expasy.org/translate/) was used to translate the nucleic acid sequence to corresponding peptide sequence and to identify a.a. changes, while BLAST (http://blast.ncbi.nlm.nih.gov/Blast.cgi) was used to find similarities between sequences from other species and *C. cardunculus*. Sequence alignments were performed using ClustalW2 (http://www.ebi.ac.uk/Tools/msa/clustalw2/).

To further predict the SNP influence at protein structure level, sequences of all haplotypes were analysed with bioinformatics tools. PredictProtein (https://www.predictprotein.org) and InterPro tools (http://www.ebi.ac.uk/interpro/) were used to identify protein domains. To predict SNP influence in post-translational modifications (PTM) several programs were used, namely for phosphorylation, SUMOylation, ubiquitination and methylation: NetPhos 2.0 [8], SUMOplot™, UbPred [71] and UbiProber [13] and MeMo 2.0 [12] were used, respectively.

### *Cc* leaf phenolic-derived extractions

A Soxhlet extraction was performed from leaves of the 20 individuals representative of haplotypes diversity (indicated in grey in Additional file 4a and b) according to Ramos et al. [72]. Before extraction, the samples were freeze-dried and ground to a granulometry of 40–60 mesh. Each sample (1.5–4 g of dry weight) was Soxhlet extracted with dichloromethane (EMPLURA) for 7 h to remove the lipophilic fraction. Two grams of the dry leftover solid residue were further extracted with 200 mL of methanol/water/acetic acid (49.5:49.5:1) under constant stirring, protected from light for 24 h at room temperature (RT). The liquid extract was then filtered; methanol and acetic acid were removed by low pressure evaporation (37–40 °C) and water by freeze-drying. The extraction yield was determined as the percentage of dry biomass material obtained. The obtained extracts were kept at RT protected from light and processed for the different analyses described below. One extract was prepared from each individual plant.

### Qualitative composition analysis of *Cc* leaf phenolic-derived extracts by HT-UHPLC-MS^*n*^

A HT-UHPLC-MS^*n*^ analysis was performed following Ramos et al. [72]. The HPLC system was coupled to a LCQ Fleet ion trap mass spectrometer (ThermoFinnigan, San Jose, CA, USA), equipped with an electrospray ionization (ESI) source. The ESI-MS was operated under the negative ionization mode with a spray voltage of 5 kV and capillary temperature of 360 °C. The flow rates of nitrogen sheath and auxiliary gas were 50 and 10 (arbitrary units), respectively. The capillary and tube lens voltages were set at −28 and −115 V, respectively. CID–MS^*n*^ experiments were performed on mass-selected precursor ions in the range of *m/z* 100–2000. The isolation width of precursor ions was 1.0 mass units. The scan time was equal to 100 ms and the collision energy was 35%, using helium as collision gas. Phenolic-derived extracts were diluted in methanol/water (50:50) (12 mg/mL) and filtered through cellulose acetate filter, 0.22 μm pore size (Millipore, USA), prior to injection. The data acquisition was carried out by using Xcalibur® data system (ThermoFinnigan, San Jose, CA, USA).

### Chlorogenic acid quantification by HPLC

CGA was quantified by HPLC. A Merck Hitachi LaChrome equipment with a L7000 interface module, a L7200 autosampler, a L7490 RI detector, a UV detector, a L7350 column oven and a L7100 pump, was used associated with the D-7000 HSM software. A reversed-phase C18 column (5 μm, 250 × 4.6 mm, Waters) at RT was used with a flow rate of 0,9 mL/min. The mobile phase consisted of A: water/acetic acid (19:1) and B: methanol (0 min: 95%, 3 min: 85%, 13 min: 75%, 25 min: 70%, 27 min: 40%, 28 min: 0%, 30 min: 95% and 38 min: 95% of A). CGA was detected at 320 nm and eluted at 15 min retention time. CGA was quantified in *Cc* leaf phenolic-derived extracts from the linear equation (*y* = 122,049 × − 1,236,603 where *x* and *y* represent CGA concentration and peak area, respectively; r^2^ = 0.999), prepared with the injection of CGA pure standard (Sigma, USA) in methanol/water (50:50) (10–800 μg/mL). *Cc* leaf phenolic-derived extracts were diluted in methanol/water (50:50) (10 mg/mL) in duplicate and filtered through cellulose acetate filter, 0.22 μm pore size, prior to injection. The data acquisition was carried out using the D-7000 HSM software (Hitachi, Japan).

### Antioxidant activity

Antioxidant activity was determined by 2,2-diphenyl-1-picrylhydrazyl (DPPH) assay according to Sánchez-Moreno et al. [75]. To determine the antioxidant activity, *Cc* leaf phenolic-derived extracts were diluted in MiliQ water (25 mg/mL) and filtered through cellulose acetate filter, 0.22 μm pore size. An aliquot of 150 μL of DPPH (100 μM) (Sigma, USA) was added to 17 μL of different *Cc* leaf phenolic-derived extract concentrations (0,10-12 mg/mL diluted in ethanol, in duplicate) in a 96-well plate. The absorbance at 520 nm was measured during 60 min against 167 μL of DPPH and 167 μL of ethanol in a Multiskan FC microplate reader (Thermo Scientific, USA). The inhibition of DPPH was calculated using the following formula:

$$ \left[\frac{\left( Abs\  DPPH- Abs\  EtOH\right)-\left( Abs\  Sample- Abs\  EtOH\right)}{Abs\  DPPH- Abs\  EtOH}\right] \times 100\% $$

IC_50_ was calculated from plotting % of DPPH inhibited and each correspondent extract concentration in logarithm. IC_50_ was also calculated on standard antioxidant compounds: butylated hydroxyanisole (BHA), ascorbic acid and CGA. Antioxidant activity index (AAI) was also calculated according to Scherer & Godoy [76]: AAI = final concentration of DPPH (μg/mL)/IC50 (μg/mL), using the DPPH final concentration of 35.45 μg/mL.

### Antithrombin activity

Antithrombin activity was measured according to Chistokhodo et al. [15]. Extracts were diluted in miliQ water (25 mg/mL) and filtered through sterile cellulose acetate filter, 0.22 μm pore size. A 96-well plate was used and 50 μL of extract (or MiliQ water as control) with 25 μL of the thrombin solution (500 units) (Sigma, USA) was placed in each well. The plate was incubated at 37 °C, and after 5 min, 50 μL of thrombin generation chromogenic substrate 2 mM (β-Ala-Gly-Arg *p*-nitroanilide diacetate) (Sigma, USA) was placed in each well. The absorbance at 405 nm was measured during 5 min in a Multiskan FC microplate reader (Thermo Scientific, USA). The percent activity was calculated using the following formula:

$$ A\%=\left[1-\frac{Vmax\  sample}{Vmax\  control}\right]\times 100\% $$

The antithrombin activity was measured in duplicate with *Cc* leaf phenolic-derived extracts at 1 mg extract/mL. CGA pure standard was alto tested between 4 and 300 μg extract/mL.

### Statistical analysis

All parameters measured were analysed using the PROC GLM (procedure general linear model) option of SAS (Statistical Analysis System, SAS Institute Inc., Cary, NC). Where differences existed, the source of the differences at a *P* < 0.05 of significance level was identified by all pairwise multiple comparison procedure. The Tukey’s test was used for pairwise comparisons.

### SNPs-phenotype and phenotype-phenotype association evaluation

For association analysis between genotype-phenotype, the C3′H and HQT coding sequences with, respectively, 3 and 8 SNPs of the 20 *Cc* plants representative of the haplotypes diversity were used (Additional file 4 a and b). Association analysis between the CGA content, biological activities and the SNPs present in target genes was performed using TASSEL 5.0 software (http://tassel.bitbucket.org) [9] with the statistical standard GLM (general linear model). Polymorphic sites carrying rare alleles (frequencies <2%) were discarded to avoid biased associations. Significant associations were accepted with *p*-value ≤ 0.05 threshold.

To correlate phenotype-phenotype (traits) significances, a Pearson correlation between CGA and biological activities was performed using SAS (SAS Institute, Inc., Cary, NC, USA).

## Results

### Haplotypes identified for *C3′H* and *HQT* genes in *C. cardunculus* from different origins

**Fig. 1:**
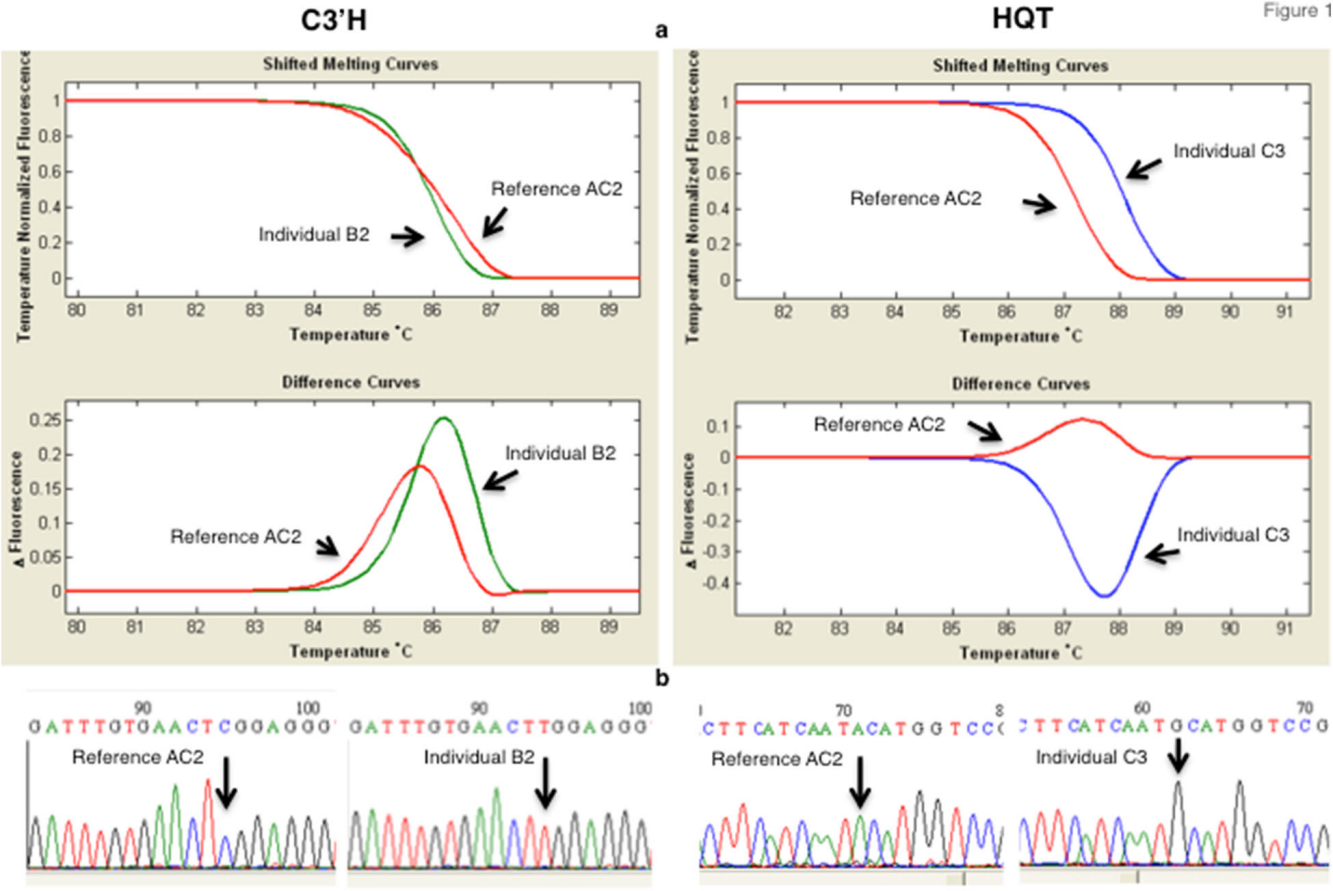
Example of one SNP identified on each C3′H and HQT amplicons by HRM curve profiles (**a**) and confirmed by sequencing (**b**). Results were obtained from the amplification of the C3′H exon 2 with primers C3H-3F and C3H-3R and from the amplification of HQT exon 2 with primers HQT-2F and HQT-2R. Reference ecotype from Beja AC2 (*red*) and B2 (*green*) and C3 (*blue*) ecotypes from Spain and Moldavia, respectively. The melting curve profiles of the reference AC2 (*red*) is different from the individual B2 (*green*) and C3 (*blue*), on C3′H and HQT genes, respectively. Sequencing of a C3′H amplicon indicates, at position 581 (exon 2), a cytosine in the reference AC2 and a thymine in B2 individual. Sequencing of a HQT amplicon indicates, at position 499, an adenine in the reference AC2 and a guanine in C3 individual

The *C3′H* and *HQT* genes were screened for point mutations in 127 *Cc* individuals from different origins (Additional file 1). HRM analysis allowed identification of different melting curves that, after sequencing, revealed 12 and 34 SNPs in *C3′H* and *HQT* coding sequences, respectively (Additional file 2). Single, double and multiple SNPs were identified in the amplicons covering the coding sequences of both genes. From these SNPs, we focused only on the nonsynonymous ones, namely 3 in *C3′H* and 8 in *HQT* (Table 1) that derived, respectively, in 4 and 17 groups of individuals with identical sequence named haplotypes (phased genotypes obtained with SNiPlay pipeline) (Table 2). All the haplotypes and SNPs identified are described, respectively, in Additional file 4(a, b) and Additional file 5. Figure 1 represents examples of SNPs identified in C3′H and HQT amplicons by HRM profiles and confirmed by sequencing.

**Table 1:** Nonsynonymous SNPs identified in C3′H and HQT genes assuming AC2 as the reference variety

	SNP change and position	A.a. change and position	SNP frequency (%)	Major allele	Minor allele	Homozygotes major allele	Homozygotes minor allele	Heterozygotes
C3′H	C581T	S194L	16.9	C	T	101	17	9
	A592C	M198L	26.0	A	C	73	12	42
	G1054A	V352I	0.4	G	A	126	0	1
HQT	A499G	T167A	39.4	A	G	49	22	56
	A547G	I183V	39.4	A	G	49	22	56
	G586A	A196T	2.4	G	A	121	0	6
	C653T	S218F	43.7	C	T	52	36	39
	G682A	A228T	37.0	G	A	67	34	26
	A802G	K268E	11.4	G	A	112	14	1
	G964A	D322N	4.7	G	A	118	3	6
	G986T	S329I	10.2	G	T	101	0	26

**Table 2:** Haplotypes identified in C3′H and HQT genes

	Haplotype	Haplotype sequence	Haplotype frequency (%)
C3′H	A	C…A…G	57.1
	B	C…C…A	0.4
	C	T…A…G	16.9
	D	C…C…G	25.6
HQT	A	A…A…G…C…G…A…G…G	2.0
	B	A…A…G…T…G…A…G…G	2.0
	C	A…A…G…T…G…G…G…G	38.2
	D	A…A…G…C…A…G…A…G	1.2
	E	G…G…G…C…A…G…G…T	5.9
	F	G…G…A…C…A…G…G…G	2.4
	G	G…G…G…C…A…G…G…G	10.2
	H	G…G…G…C…A…A…G…G	0.4
	I	G…G…G…C…G…A…G…G	3.9
	J	A…A…G…C…A…A…G…G	1.6
	K	A…A…G…C…A…G…G…G	10.2
	L	G…G…G…C…A…A…G…T	1.6
	M	A…A…G…T…G…G…G…T	2.8
	N	A…A…G…C…G…G…G…G	2.8
	O	G…G…G…C…G…G…G…G	10.6
	P	G…G…G…C…A…G…A…G	3.5
	Q	G…G…G…T…G…G…G…G	0.8

**Fig. 2:**
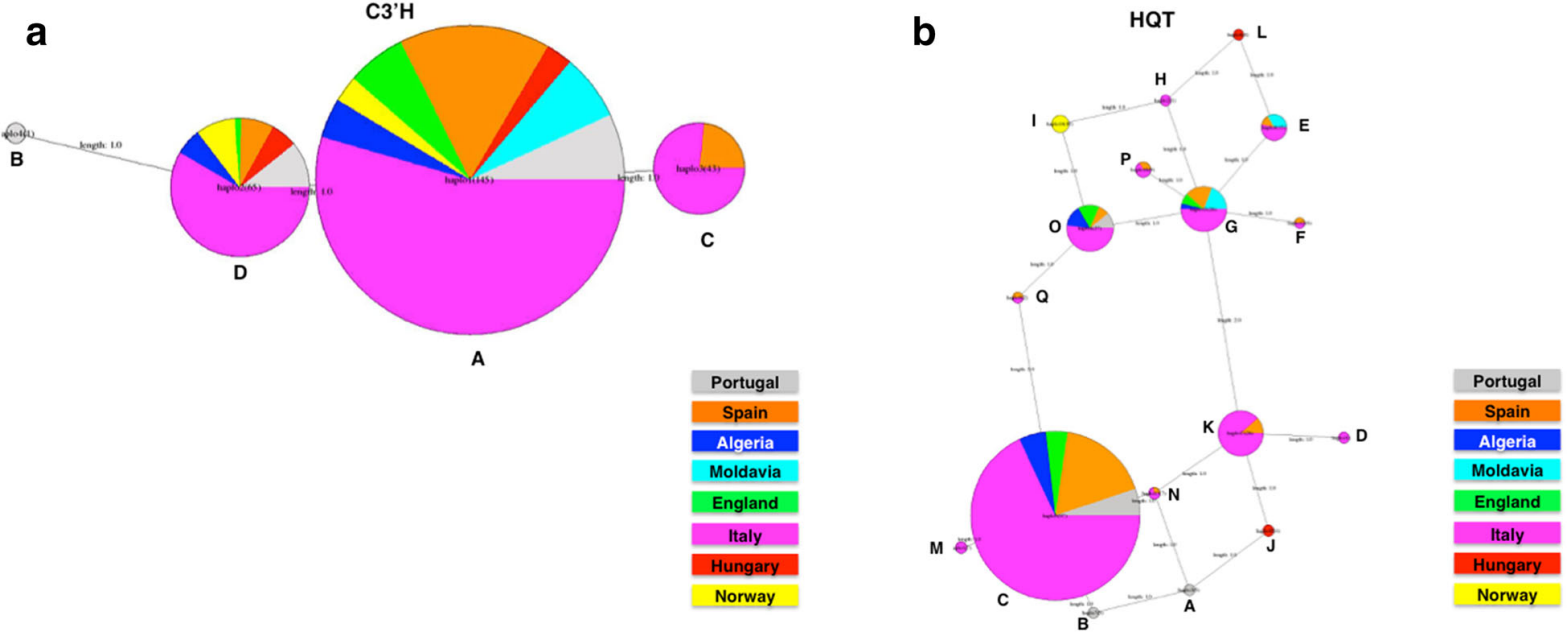
Distribution of the observed haplotypes in C3′H (**a**) and HQT (**b**) genes. The size of the *circles* is proportional to haplotype frequency, the length of the *lines* is proportional to the number of mutation steps between genotypes and their proximal states, and *pie charts* indicate the contribution of plant origin groups to a particular haplotype

Network analysis of different haplogroups was constructed based on all the SNPs found in coding regions for both genes to infer relationships between haplotypes (Fig. 2a and b). Haplotype A of *C3′H* gene is the dominant haplotype while haplotype B is only represented by a single individual. Haplotypes A and D of this gene present higher diversity within all the origins represented. Haplotype C of *HQT* gene is the dominant one among the analysed populations. From the 8 studied regions, haplotypes C, O and G of this gene were always present in 5 regions - Algeria, Italy, Spain, England and Portugal or Moldavia. All individuals from Norway were homozygous and fall within haplotype I of *HQT* gene. The four individuals from Hungary (heterozygous) comprise the two haplotypes J and L, while the five individuals of the studied Portuguese cultivated variety were heterozygous and comprise haplotypes A and B of *HQT* gene.

### Changes at the protein level

We focused on the SNPs present in coding regions and therefore we decided to further examine changes in amino acid residues assessing their putative influence on protein structure as compared to the reference individual/allele AC2. AC2 was heterozygous for both *C3′H* and *HQT* genes.

### Putative C3′H protein changes

**Fig. 3:**
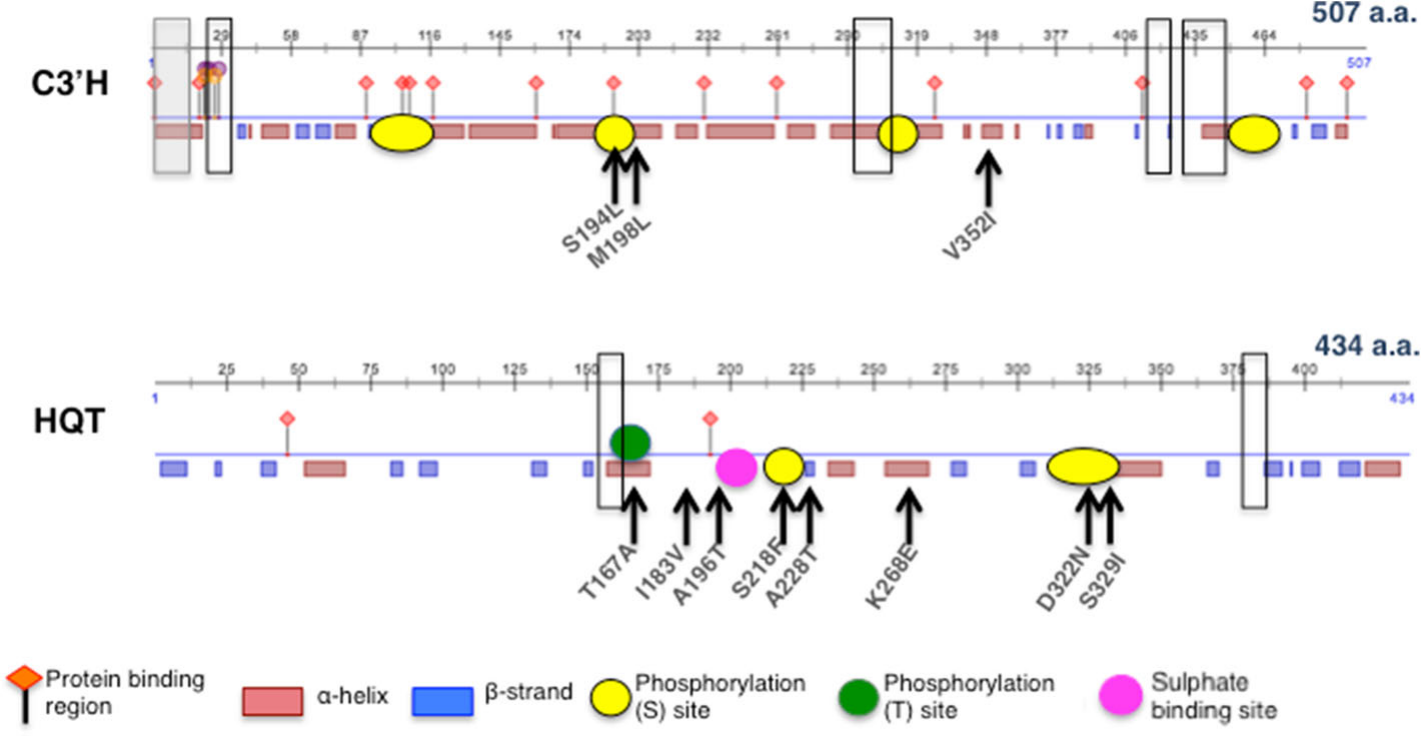
Schematic representation of the C3′H and HQT reference aminoacid sequences (haplotype A). Domains were identified with PredictProtein and InterPro tools. The serine (S) and threonine-rich (T) sites were predicted based on phosphorylation probability obtained with NetPhos 2.0. The ubiquitination sites (K) were predicted by UbPred and UbProber. The *upperline* represent the aminoacid position. The conserved domains representative of P450 family in C3′H and the acyltransferase family in HQT are surrounded in *black boxes. Filled grey boxes* indicate the signal peptide domain in C3′H. SNP positions are indicated with *arrows* indicating the residue of the reference AC2_1 and the substituted residue

Bioinformatic tools such as InterPro and PredictProtein were used to analyse the position of these SNPs in C3′H protein allowing the identification of putative structural/functional protein domains. This analysis revealed that C3′H is a member of the cytochrome P450 family, an oxireductase acting as monooxygenase (InterPro). The alignment of C3′H protein with P450 proteins from *Coffea canephora* (ABB83677), *Arabidopsis thaliana* (NP850337), *Sorghum bicolor* (O48956), *Salvia miltiorrhiza* (ACA64048), *Populus tomentosa* (AFZ78540) and *Jatropha curcas* (XP_012075888) revealed identities between 70 and 88%, and allowed the identification of P450 conserved domains. Schematic representation of C3′H aminoacid sequence is represented in Fig. 3. According to Phobius [40] and SignalP 4.1 [68] prediction tools, C3′H protein has a putative signal peptide on the first 18 residues on N-terminal, while the last 489 protein residues are predicted to be non-cytoplasmic. Predotar (https://urgi.versailles.inra.fr/Tools/Predotar) and LocTree3 [32] tools confirmed the endoplasmic reticulum localization of the C3′H protein. The reference individual AC2 is heterozygous for *C3′H* gene at nucleotide positions 592 and 1054, and shows two haplotypes, A and B, according to Phase and Gevalt algorithms (Additional file 4a). As haplotype B is rare (<1%) and only composed by this variant of the reference individual, all the SNPs analysed were compared relative to the most probable haplotype of this individual, haplotype A. The M198L polymorphism (methionine to leucine) (Table 1), which occurs in haplotypes B and D relative to reference haplotype A, occurs in the transition zone between a random coil protein organization and on a α-helix conformation. Furthermore, NetSurfP 1.1 predicted that leucine or methionine in position 198 are buried between the two adjacent residues that are solvent exposed. Since both amino acids are classified as nonpolar, our hypothesis is that the straight side chain of methionine with a *S*-methyl thioether at the γ-carbon could alter the hydrophobicity causing protein destabilization as indicated by Lipscomb et al. [47]. The V352I (valine to isoleucine) alteration occurs only in haplotype B and could have lower impact on the protein secondary structure as both residues are very similar and the SNP occurs at a region predicted to be mostly in a buried α-helical zone. Moreover both residues were not susceptible to post-translational modifications. Haplotype C is unique in carrying the S194L mutation (serine to leucine). According to InterPro and PredictProtein tools, this alteration occurs in a predicted random coil solvent exposed zone of the protein, as well as in a protein-binding region (S194 and E195). The putative impact of SNPs changes in the predicted protein secondary structure was also evaluated at the level of post-translational modifications such as phosphorylation, ubiquitination, SUMOylation and methylation. The serine-to-leucine substitution in residue 194 could imply loss of a residue putatively phosphorylated. The possibility that these changes account for the alteration of a polar to a hydrophobic residue, could lead to protein conformational changes and functional modification. Compared to reference haplotype A, haplotype C of *C3′H* (Fig. 3) presents two SNPs identified as M198L and V352I while haplotypes C and D carry only the S194L and M198L mutations, respectively.

### Putative HQT protein changes

The hydroxycinnamoyl HQT protein from *Cc* should be accumulating in the cytoplasm (Predotar tool). The alignment of HQT sequences belonging to the plant hydrocinnamoyl transferase family from *Ipomoea batatas* (BAA87043), *Nicotiana tabacum* (CAE46932), *Solanum lycopersicum* (CAE46933) and *Theobroma cacao* (XP_007023475) revealed identities between 70 and 80% and indicated the existence of two structural motifs, HXXXD and DFGWG, highly conserved in the BAHD acyltransferase family. The HXXXD domain lies in the central protein portion and the histidine (H153) residue is necessary for full enzymatic activity [81]. The DFGWG motif, located near the protein C-terminus, is important for catalytic activity or binding to coenzyme A [6, 82]. The schematic representation of HQT aminoacid sequence is represented in Fig. 3. The reference individual AC2 is heterozygous in nucleotide position 653 and, according to Phase and Gevalt algorithms, could present the two haplotypes A and B (Additional file 4b) that encode respectively, a serine (S) or a phenylalanine (F) in residue 218. Both haplotypes occur at the same frequency (2%) but in the studied population nucleotide position 653, the most prevalent allele, codes for cytosine (frequency of 56.3%), as it happens in haplotype A, which was chosen as the reference haplotype. This S218F alteration, relative to haplotype A, occurs in haplotypes B, C, M and Q in a predicted protein random coil solvent-exposed zone. Serine is a polar residue while phenylalanine is hydrophobic and this difference could have an effect on protein activity. Additionally, the high phosphorylation probability of the S218 is no longer expected in F218, with a putative impact on protein function. The T167A modification (threonine to alanine) occurs in haplotypes E, F, G, H, I, L, O, P and Q in a predicted buried α-helix region of the protein. The alteration from a polar threonine to a hydrophobic alanine, along with loss of a predicted phosphorylation site (T167), could affect HQT protein conformation and the stability of these haplotypes. The I183V alteration (isoleucine to valine) is present in the same haplotypes as the previous mutation T167A, and occurs in a buried random coil protein zone. Given the chemical and structural similarity of both residues this change may have a low impact on the overall protein structure/function. The A196T alteration (alanine to threonine) that occurs only on haplotype F could have a high impact in the HQT function. This alteration occurs near a sulphate-binding region (E199) and the change from a hydrophobic to a polar residue in the solvent exposed-zone could therefore affect protein structure, and increase chances of threonine phosphorylation. Haplotype F is composed by 50% accessions from Italy and 50% from Spain. The A228T change present in haplotypes D, E, F, G, H, J, K, L and P leads to an alteration from a hydrophobic alanine to a polar threonine in a predicted buried β-strand zone of the protein that could affect its conformation and stability. The alteration observed in 11 haplotypes (Additional file 4b) from lysine to glutamate (K268E) is predicted to be solvent exposed and occurs between two buried adjacent residues in a protein α-helix zone. Although, glutamate (E) residues can be methylated [80], available bioinformatics tools are only available for lysine (K) and arginine (R) residues. Glutamate (K268) it was not predicted to be a target for PTM. Taking this information into account plus the fact that both K and E residues are predicted to be solvent exposed, a change in a positive (K) to negative (E) local charge is likely to affect protein function. The D322N modification (aspartate to asparagine) observed in haplotypes D and P also occurs in a solvent exposed site between two buried adjacent residues in a protein α-helix zone. Although these residues are not susceptible to phosphorylation or ubiquitination, the N322 modification in haplotypes D and P reduced the chances for PTM at residues S319, T323 and K333. If these are indeed sites for HQT regulation by phosphorylation and ubiquitination, the D to N alteration reduces the PTM probability, with putative impact on protein function. Regarding the serine to isoleucine (S329I) modification in haplotypes E, L and M of HQT, it occurs in a predicted α-helix exposed zone, which could account for a modified polarity. The change of a serine residue could also account for a loss of PTM, with a putative implication in protein function.

### Extraction yield of *Cc* leaf phenolic extracts

The extraction yield determined in 20 methanol/water/acetic acid (49.5:49.5:1) extracts representative of *Cc* haplotype diversity are presented in Table 3. The extraction yields of the *Cc* leaf varied between 6.0% (AC2) and 47.0% (K3 and M1) (7.8-fold) (Table 2). With the exception of AC2 (6.0%), D4 (6.5%) and A4 (19.3%) individuals, the extraction yields were slightly higher than those previously reported in the literature for methanol extracts of cardoon leaves (34.72%) [23] and for methanol/water/acetic acid extracts of cultivated cardoon leaves (28.0%) [72], the same ecotype of AC2 reference individual. Since the *Cc* plants used in this study were maintained, stored and handled in identical conditions, genetic differences could explain extraction yield differences, especially for the lower yields obtained for AC2 and D4 individuals. Yield differences obtained when comparing this study with others in the literature could not only be associated with the extraction procedures, but also with the different geographic location, life cycle stage, age and collection year.

**Table 3:** Extraction yields, CGA content, DPPH scavenging effect and antithrombin activity of leaf phenolic-derived extracts from the 20 *Cc* individuals

Individual	Extraction yield (% *w/w*)	CGA content (% *w/w* extract)	DPPH scavenging effect IC_50_ (μg/mL)	Antioxidant activity index (AAI)	Antithrombin activity (%)
A1	45.5	0.14 ± 0.00^kl^	1154.19 ± 53.05^b^	0.03	21.63 ± 0.31^fg^
A4	19.3	0.25 ± 0.02^ij^	351.70 ± 54.22^fg^	0.10	41.16 ± 5.84^ab^
B3	40.0	0.15 ± 0.00^kl^	466.88 ± 23.56^cd^	0.08	42.85 ± 5.03^a^
C1	42.5	0.30 ± 0.00^gh^	386.46 ± 22.312^defg^	0.09	41.18 ± 5.50^ab^
D4	6.5	0.23 ± 0.00^j^	448.80 ± 5.03^de^	0.08	31.22 ± 1.07^cd^
E4	41.0	0.78 ± 0.00^c^	311.67 ± 6.45^gh^	0.11	29.13 ± 1.60^de^
G2	40.5	0.51 ± 0.00^e^	159.14 ± 2.11^ij^	0.22	21.48 ± 2.50^fg^
I2	41.7	0.36 ± 0.02^f^	209.56 ± 10.06^i^	0.17	37.07 ± 2.71^abc^
J3	39.0	0.75 ± 0.05^c^	122.66 ± 7.09^jk^	0.29	25.77 ± 4.05^def^
J4	29.9	0.84 ± 0.07^b^	114.69 ± 0.51^jk^	0.31	31.66 ± 2.13^cd^
J5	38.5	1.19 ± 0.04^a^	47.22 ± 1.80^k^	0.75	15.69 ± 1.37^gh^
K3	47.0	0.30 ± 0.00^hi^	232.30 ± 8.45^hi^	0.15	28.44 ± 2.86^de^
M1	47.0	0.34 ± 0.00^gh^	533.65 ± 64.87^c^	0.07	36.17 ± 1.37^bc^
O4	30.2	0.37 ± 0.03^f^	210.85 ± 11.59^i^	0.17	32.11 ± 0.59^cd^
Q5	37.0	0.65 ± 0.02^d^	44.48 ± 1.09^k^	0.80	24.74 ± 4.68^ef^
Y1	40.0	0.11 ± 0.00^l^	1417.64 ± 66.63^a^	0.03	14.95 ± 4.21^h^
Z3	43.6	0.18 ± 0.01^k^	432.60 ± 87.52^def^	0.08	24.45 ± 0.58^ef^
AA1	41.8	0.25 ± 0.00^ij^	459.22 ± 84.88^cde^	0.08	27.04 ± 0.77^def^
AB1	40.0	0.24 ± 0.03^j^	380.39 ± 32.17^efg^	0.09	24.82 ± 1.09^ef^
AC2	6.0	0.35 ± 0.01^fg^	152.20 ± 7.44^ij^	0.23	22.98 ± 2.52^ef^
CGA	-	-	5.24 ± 0.35	6.77	59.20 ± 0.36
BHA	-	-	7.27 ± 0.13	4.88	-
Ascorbic acid	-	-	2.38 ± 0.09	14.89	-

*CGA* chlorogenic acid, *BHA* butylated hydroxyanisole

Results correspond to the mean ± standard deviation estimated from two determinations. Means with different letters within the same column are statistically different (*P* < 0.05)

### Major phenolic compounds identified in *Cc* leaf phenolic extracts

Phenolic compounds were identified in the methanol/water/acetic acid extracts of *Cc* leaves by HT-UHPLC-UV-MS^*n*^ analysis. Table 4 shows the retention time, the maxima UV wavelengths, and the [M-H]^−^ ion of main phenolic compounds identified in the *Cc* leaf phenolic-derived extracts. These compounds were identified by comparing these data with those obtained by Ramos et al. [72]. Two hydroxycinnamic acids were identified in the *Cc* extracts, named as CGA and 1,5-di-O-caffeoylquinic acid, based on the UV spectra and the detection of [M-H]^−^ ions at *m/z* 353 and 515, respectively (Table 4) [72]. Two flavones, as luteolin 7-*O*-glucoside and a luteolin acetyl-hexoside isomer, were identified according with the UV spectra and the detection of [M-H]^−^ ions at *m/z* 447 and 489, respectively [72]. These phenolic compounds were found in all the methanol/water/acetic acid extracts of *Cc* leaves, derived from the individual A1 to the individual AC2 (Table 4).

**Table 4:** HT-UHPLC-UV-MS^*n*^ data of the main phenolic compounds identified in the methanol/water/acetic acid leaf extracts (49.5:49.5:1) from the 20 *Cc* individuals (A1 to AC2)

Compound	RT (min)	UV λ_max_ (nm)	[M-H]^−^ (m/z)
5-*O*-Caffeoylquinic acid	5.08	244, 296, 324	353
Luteolin 7-*O*-glucoside	15.84	254, 261, 267, 347	447
1,5-Di-*O*-caffeoylquinic acid	17.70	244, 299, 327	515
Luteolin acetyl-hexoside	21.07	254, 266, 347	489

### Chlorogenic acid content, antioxidant and antithrombin activities

The CGA content, as well as antioxidant and antithrombin activities were determined in the 20 phenolic-derived extracts of *Cc* leaves and the results are presented in Table 3.

Regarding the CGA content (Table 3), *Cc* leaf extracts prepared from the J5 individual showed the highest CGA abundance, accounting for 1.19% (*w/w* extract) (*P* < 0.05). The CGA content of this extract was 8.5-fold higher than the lowest one in the Y1 individual with 0.11% (*w/w*). CGA levels of the analysed *Cc* leaf phenolic extracts were slightly higher than the one earlier described for cultivated cardoon leaf extracts (0.01% *w/w*) [72], including that for the same ecotype as the AC2 reference individual (0.35%). This difference could not only be related to a different life cycle stage and collection year but also to the age of the plant material analysed since, in this work, the CGA level was quantified in 9-month-old plants, while Ramos et al. [72] determined it in adult plants (>5 years).

Phenolic compounds present in medicinal plants are well-known for their antioxidant properties [91]. Scavenging activities of antioxidant agents are thought to be highly important in preventing damaging actions of free radicals involved in cardiovascular diseases and cancer [49]. In the present work, the antioxidant activity of *Cc* leaf phenolic-derived extracts was assessed through their scavenging capacity against DPPH free radicals. The inhibitory concentrations of *Cc* extracts and standard antioxidants needed to decrease by 50% (IC_50_) the initial concentration of DPPH were thus determined (Table 3). The lower the IC_50_ the higher the antioxidant power. IC_50_ values, regarding DPPH scavenging capacity of *Cc* extracts ranged from 44.48 μg/mL to 1417.64 μg/mL in the Q5 and Y1 individuals, respectively, with a 31.9-fold difference (*P* < 0.05). Nevertheless, *Cc* leaf phenolic-derived extracts were less active relatively to the tested standard antioxidants, namely BHA (IC_50_ = 7.27 ± 0.13 μg/mL) and ascorbic acid (IC_50_ = 2.38 ± 0.09 μg/mL). The IC_50_ values of *Cc* extracts were also higher than CGA (IC_50_ = 5.24 ± 0.35 μg/mL). Comparing values in terms of molarity, CGA IC_50_ (0.014 ± 0.000 μM) is similar to the standard antioxidant ascorbic acid (0.013 ± 0.000 μM) and lower than BHA (0.040 ± 0.000 μM) showing an advantageous antioxidant potential for CGA present in *Cc* extracts. In order to compare the antioxidant efficiency of these extracts with data reported in the literature, the antioxidant activity index (AAI) was calculated taking the final concentration of DPPH into account (35.45 μg/mL). AAI of *Cc* leaf phenolic-derived extracts from the Q5 and J5 individuals (0.80 and 0.75, respectively) are in the same range as those obtained in previous studies using cardoon leaf extracts [23] and higher than those obtained for cultivated cardoon leaf extracts (0.14) [72]. Even for the reference individual AC2, the same ecotype previously studied by our research team [72], a higher AAI was also observed in the present work (0.23) which could be related to the higher CGA levels observed herein and explained above. Given the ability of CGA to scavenge DPPH free radicals, fractionation methodologies should be developed for hydroxycinnamic acids, or other phenolic compounds from *Cc* (especially for Q5 and J5 individuals), for potential use as alternative antioxidants in the food industry. Previous experiments from our research team using nanofiltration resulted in total phenolic compounds concentration, as well on chlorogenic acid levels improving the antioxidant activity in *C. cardunculus* leaf extracts [10].

Thrombin is an important enzyme in the blood coagulation process, being responsible for the conversion of soluble fibrinogen into stabilized insoluble fibrin. Its inhibition is important against many blood coagulation and platelet disorders [15]. Considering that 50% of the patients with tumours or cardiac irregularities such as atrial fibrillation undergo thrombosis and 95% show clotting activation [54], the effective inhibition of thrombus development can be reached by using selective thrombin inhibitors with no effect on any other coagulant enzyme [15]. Within this context, there is currently a quest for identification of novel thrombin inhibitors. Thus the *Cc* leaf phenolic extracts from all 20 individuals (at 1 mg/mL) were tested for their thrombin inhibitory potential (Table 3). The inhibition levels detected varied from 14.95 to 42.85%, respectively, in the Y1 and B3 individuals (2.9-fold). The antithrombin effect of B3 was not statistically different from those of C1 and A4 *Cc* extracts (41.18 and 41.16%, respectively) (*P* > 0.05). Results from other studies using methanol extracts (in the same extract concentration) of *Melia azedarach*, *Cyperus globulosus*, *Cinnamomum camphora*, *Ambrosia artemisiifolia*, *Paspalum dilatatum* and *Woodwardia radicans*, revealed similar percentages of thrombin inhibition (≈40%). However, higher percentages of antithrombin activity (≈80–90%) were also described, namely with *Callistemon lanceolatus*, *Lagerstroemia indica*, *Myrica cerifera*, *Polytrichum commune* and *Calocasia esculenta* [15, 54].

Interestingly, CGA standard (at 300 μg/mL), also showed thrombin inhibition activity (59.2%), in contrast to what was previously described by Bijak et al. [7], who reported that CGA had no significant inhibitory effect on thrombin activity at ≈354 μg/mL.

### Association and correlation analysis

For SNPs phenotype association analysis we used CGA content, antioxidant and antithrombin activities (Table 3) of the 20 individuals representative of all the allelic variants found at C3′H and HQT coding sequences (Additional file 4a, b). The association analysis on TASSEL software identified 3 significant associations between SNPs and the studied traits with the statistical standard GLM model, minor allele frequency (MAF > 2%) and *p*-value ≤ 0.05 (Table 5). The SNPs present in the *C3′H* gene did not reveal any association with traits. The two SNPs (S218F and D322N) from the *HQT* gene showed significant associations with CGA content and antioxidant activity.

**Table 5:** Significant associations (*p* ≤ 0.05) identified between SNPs positions and studied traits

Gene	SNP position	Aa residue change	Protein change influence	CGA content	Antioxidant activity	Antithrombin activity
HQT	C653T	S218F	From a polar to a hydrophobic residue. Serine phosphorylation site loss.	✓	✓	-
	G964A	D322N	From a negative to a polar residue. Influence the 319 and 323 phosphorylation sites and 333 ubiquitination site		✓	-

(✓): Significant association (−): No association

To correlate trait significances, a Pearson correlation between results of CGA, antioxidant and antithrombin activities was further performed. This study revealed an inversely significant association with CGA content and IC_50_ (*P* = 0.005 and *r* = −0.6). Antithrombin activity, however, was not related to any SNP or trait.

## Discussion

This work characterizes two *Cynara cardunculus* genes, related to chlorogenic acid biosynthesis. A high-resolution melting approach was used to genotype single nucleotide polymorphisms with the objective of identifying putative variants in the corresponding enzymes. This method has been frequently used in clinical chemistry, patient genotyping and human pathology since 2003 [87, 89]. Recently, it has also been used in genetic variability studies, mutation screens and genotyping in plants such as apple [11], barley [46], grapevine [53], almond [90], olive [64], pepper [39], sweet cherry [29], peach [14], artichoke [77], durum wheat [63] and rice [83].

A collection of 29 accessions comprising 127 individuals of *Cynara cardunculus* was used for HRM analysis. Considering the genetic variability of *Cc*, a diploid cross-pollinated species [55], whenever possible, 5 individuals from the same accession were selected. Most accessions analysed were from wild cardoon (25 accessions, 109 individuals). In addition, accessions of cultivated cardoon and artichoke were also analysed. In the context of germplasm origin, Italy was the best-represented country, with accessions available from different regions.

Considering the advantages of the HRM technique, such as the possibility of evaluating a large sample number (avoiding sequencing all of them), its simplicity, robustness and fast technique [38, 41], this method proved to be, in our work, quite efficient in discriminating DNA with only one base difference and to allow distinction between heterozygous/homozygous SNPs in the amplicons of *C3′H* and *HQT* genes. Variability was observed by the heterozygosity level obtained. HRM allowed identification of 46 SNPs (confirmed by sequencing) in the coding sequences of *C3′H* and *HQT* genes. From these SNPs, 35 were classified as synonymous and, since they locate within exonic regions expected to be conserved in mature mRNA and consequently not interfering with mRNA splicing, they were no longer discussed within the present study.

For diploid species, such as *Cynara*, multiple statistical algorithms are used to infer phase genotypes. To reconstruct haplotypes of nonsynonymous SNPs, we used the SNiPlay pipeline that employs Phase or Gevalt algorithms. This analysis is based on identification of sets of alleles that are found together in multiple individuals and allowed, in our study, the definition of 21 haplotypes. The *C3′H* gene proved to be very conserved, with only 4 haplotypes, while for *HQT*, 17 haplotypes were identified. These results confirm HRM as good strategy to discover possible new allelic variants to genotype plants for assisted breeding.

The isolation and mapping of the *C3′H* genomic sequence from *Cynara cardunculus* L. var. *scolymus* was reported by Moglia et al. [61] with a nucleotide difference at position 447 (nucleotide not identified). The *HQT* cDNA sequences of artichoke and cultivated cardoon were first described by Comino et al. [17] and a G834C alteration was reported in both sequences. Sonnante et al. [77] also identified T486C and G271A alterations in *C3′H* and *HQT* coding sequences of artichoke, respectively. Although we could not identify these changes in our study, this work reports a number of novel SNPs in the *C3′H* and *HQT* gene sequences from different *Cynara cardunculus* individuals.

The 11 nonsynonymous SNPs identified on *C3′H* and *HQT* genes were characterized using bioinformatics tools to assess their possible influence at the protein level. The C3′H protein belongs to the CYP98 family of plant cytochromes P450 (plant metabolism related) which has been labelled as the family of enzymes performing the *meta*-hydroxylation step in the phenylpropanoid pathway. This hydroxylation is essential not only for the synthesis of chlorogenic acid but it is also important for lignin biosynthesis [61]. The chlorogenic acid synthesis pathway is not yet completely defined but it seems that C3′H could use *p*-coumaroylquinic (first route) or/and *p*-coumaroylshikimic acids (second route) as substrates. C3′H from artichoke appears to have a lower affinity for quinate esters than shikimate esters [61] and route 2 has been reported as an important way for plants to synthesize CGA [17, 51, 66, 85]. In our study, *C3′H* gene sequence analysis by bioinformatics tools revealed a putative endoplasmatic reticulum (ER) location for the C3′H protein. Phobius and SignalP tools revealed a signal peptide at the amino terminus of the protein. According to the Uniprot database, signal peptides are found in proteins that are targeted to ER and either destined to be secreted to the extracellular or periplasmic space, or to be retained in the ER lumen. Predotar confirmed the cellular ER localization of the C3′H protein although this has not been confirmed in the literature. The S194L and M198L alterations may account for changes in protein structure/function. The M198L can lead to an alteration of hydrophobicity and protein destabilization since there is evidence of an estimated loss of stability of about 1.4 kcal/mol for each leucine-to-methionine substitution at a buried site within a folded protein [47].

HQT is a hydroxycinnamoyl transferase implicated in CGA production that can use either *p*-coumaroyl-CoA or caffeoyl-CoA esters as an acyl donor, using quinic acid as an acceptor [17]. A strong support for the second route (using caffeoyl-CoA) has been provided in tomato, where *HQT* gene silencing resulted in a 98% reduction in the level of chlorogenic acid [66]. The Predotar tool predicted HQT localization within the cytoplasm, but there is no cellular information to confirm this. The T167A, A196T, S218F, A228T, K268E, D322N, S329I alterations suggest destabilization of protein conformation, thus affecting HQT protein function. The discovery of these SNPs on C3′H and HQT coding sequences offers new research approaches to the study of the phenylpropanoid pathway in *Cynara cardunculus*.

Based on the different haplotypes obtained for *C3′H* and *HQT* genes (Additional file 4a, b), 20 individuals representative of the diversity observed were selected to produce leaf phenolic-derived extracts in a search for a putative association between nucleotide variations and phenotype. Leaves were chosen to produce the phenolic-derived extracts, since it has been previously reported as the *Cc* organ with the higher extraction yields [23, 72] as well as a higher polyphenol content [23].

Association analyses are becoming increasingly important to exploit the natural diversity of genes related to a particular phenotype. The SNPs present in the *C3′H* gene did not reveal any association with traits, and the antithrombin activity was also not significantly associated with any SNP. A total of two out of eight SNPs (S218F and D322N) from the *HQT* gene showed significant associations with CGA content and antioxidant activity. As previously explained, the S218F mutation leads to an alteration of hydrophobicity (S to F residues) in a solvent-exposed protein zone and also leads to a loss in a serine phosphorylation site resulting in a putative regulatory level alteration likely to affect protein function. The S218F alteration present in haplotypes B, C, M and Q is associated both with CGA content and antioxidant activity (Table 5). As we observed (Table 3), the individuals J4, J5 and Q5 carrying this mutation (haplotypes C, M and Q) presented the highest chlorogenic acid content (not counting J3 and E4 individuals) and in turn the highest antioxidant activity (lowest IC_50_ values). Despite the protein conformation predictions explained above, it seems that the existence of the S218F alteration on these HQT haplotypes could increase CGA content. Since biological properties of plants are due to their content in different metabolites, alterations in *HQT* gene can influence, positively the final CGA content and thus the level of the antioxidant activity. This result was confirmed by the Pearson correlation, which revealed an inversely significant correlation between CGA content and IC_50_ meaning that CGA content improved the plant antioxidant activity. The D322N alteration present in haplotypes D and P could cause protein destabilization and changes at the regulatory level. This mutation leads to a change from a negative (D residue) to a polar uncharged residue (N) and influences the 319 and 323 phosphorylation sites as well as 333 ubiquitination causing loss of these regulatory elements. This alteration was significantly associated with antioxidant activity (IC_50_). Heterozygous individual Y1 (haplotype D and P) yielded the highest IC_50_ value. However, this association was not correlated with the CGA content.

Nevertheless, it should be highlighted that these putative associations could represent false positives and, since the association analysis power is highly dependent on the number of genotypes employed [4, 28], a larger population would be desirable. In addition, further studies should validate the present results by replicating and verifying the associations to include functional analysis.

Antithrombin activity was not related to any SNP or trait.

## Conclusion

In order to develop a targeted breeding strategy, directed at improving the content of the relevant plant metabolites, it is important to have a better knowledge of the *C. cardunculus* genome. Our work provides, for the first time, detailed information about the natural allelic variants of *C3′H* and *HQT* genes involved in *C. cardunculus* phenylpropanoid pathway.

The qualitative analysis in all the 20 *Cc* leaf phenolic-derived extracts, identified two hydroxycinnamic acids, CGA and 1,5-di-O-caffeoylquinic acid, as well as two flavones, luteolin 7-*O*-glucoside and luteolin acetyl-hexoside. The differences obtained in the biological activities characterized here could be related to different synergisms of the total phenolic content including flavonoids of *Cc* extracts. The phenolic content of a plant depends on a number of intrinsic (genetic) and extrinsic (environmental) factors. The genotype proved to be a major determinant of variation in *Cc* polyphenol content and profile [23, 27, 67]. In this study different polyphenolic compositions could also be associated with genotypic differences of *Cc* individuals from diverse geographic locations. Usually, antioxidant activity has been correlated with total phenolic content [86]. Although in the present work CGA content is less than 1% in *Cc* leaf extracts, we found an evident correlation between CGA content and antioxidant activity meaning that this compound could have a significant impact on overall antioxidant power. Thus further studies on quantification, isolation and characterisation of *Cc* compounds responsible for specific biological activities are highly recommended.

In the context of the molecular diversity of target genes, the association results shown in this study may provide markers that are useful for *Cc* genetics, trait selection and breeding applications. Association analysis allowed identification of interesting haplotypes, such as C, M and Q haplotypes, which present the S218F alteration on the HQT sequence, associated with higher chlorogenic acid content and improved antioxidant activity. Further, the SNPs detected herein will be interesting for future association studies with other traits. This study opens new lines for research, specifically for validation of the identified SNPs by functional analysis and correlation to particular phenotypes.

## Additional files

Additional file 1:
*Cynara cardunculus* accessions used in this study. (XLSX 22 kb)
Additional file 2:
*C3′H* and *HQT* gene structures and primer pairs (triangle arrows) designed for HRM analysis. (*) SNPs position; (#) a.a. alteration. The introns are indicated in grey. (PDF 57 kb)
Additional file 3: Primer pairs designed for HRM analysis. (XLSX 50 kb)
Additional file 4: (a) Haplotypes identified for *C3′H* gene. Individuals selected for phenotypic trait analysis are indicated in grey. (b) Haplotypes identified for *HQT* gene. Individuals selected for phenotypic trait analysis are indicated in grey. (ZIP 89 kb)
Additional file 5: SNPs identified on *C3′H* and *HQT* genes. (XLSX 44 kb)
Additional file 6: Dataset supporting the conclusions of this article. (XLSX 75 kb)
Additional file 7: Individuals C3′H sequences. (FAS 189 kb)
Additional file 8: Individuals HQT sequences. (FAS 162 kb)

## Abbreviations

AAI: Antioxidant activity index
BHA: Butylated hydroxyanisole
C3′H: *p*-coumaroyl ester 3′-hydroxylase
*Cc*: *Cynara cardunculus*
CGA: Chlorogenic acid
DPPH: 2,2-diphenyl-1-picrylhydrazyl
ESI: Electrospray ionization
GLM: General linear model
HQT: Hydroxycinnamoyl-CoA: quinate hydroxycinnamoyl transferase
HRM: High-resolution melting
PTM: Post-translational modification
RT: Room temperature
SNP: Single nucleotide polymorphism

## References

[1] Abrantes S (2007). Cynara cardunculus L. alkaline pulps: alternatives fibres for paper and paperboard production. Bioresour Technol.

[2] Acquadro A (2005). Development and characterization of microsatellite markers in Cynara cardunculus L. Genome.

[3] Adzet T, Camarasa J, Laguna JC. Hepatoprotective activity of polyphenolic compounds from Cynara scolymus against CCl4 toxicity in isolated Rat hepatocytes. J Nat Prod. 1987;50(4):612–7. Available at: http://dx.doi.org/10.1021/np50052a004. 10.1021/np50052a004 3430163

[4] Agrama HA, Eizenga GC, Yan W (2007). Association mapping of yield and its components in rice cultivars. Mol Breed.

[5] Azzini E (2007). Absorption and metabolism of bioactive molecules after oral consumption of cooked edible heads of *Cynara scolymus* L. (cultivar Violetto di Provenza) in human subjects: a pilot study. Br J Nutr.

[6] Bayer A, Ma X, Stöckigt J (2004). Acetyltransfer in natural product biosynthesis––functional cloning and molecular analysis of vinorine synthase. Bioorg Med Chem.

[7] Bijak M (2013). Antithrombin effect of polyphenol-rich extracts from black chokeberry and grape seeds. Phytother Res.

[8] Blom N, Gammeltoft S, Brunak S (1999). Sequence and structure-based prediction of eukaryotic protein phosphorylation sites. J Mol Biol.

[9] Bradbury PJ (2007). TASSEL: software for association mapping of complex traits in diverse samples. Bioinformatics.

[10] Brás T (2015). Impact of extraction parameters and concentration by nanofiltration on the recovery of phenolic compounds from Cynara cardunculus var. altilis: assessment of antioxidant activity. Ind Crop Prod.

[11] Chagné D (2008). Genomics development of a set of SNP markers present in expressed genes of the apple. Genomics.

[12] Chen H (2006). MeMo: a web tool for prediction of protein methylation modifications. Nucleic Acids Res.

[13] Chen X (2013). Incorporating key position and amino acid residue features to identify general and species-specific Ubiquitin conjugation sites. Bioinformatics.

[14] Chen Y, Wilde HD (2011). Mutation scanning of peach floral genes. BMC Plant Biol.

[15] Chistokhodo N, et al. Antithrombin activity of medicinal plants from central Florida. 2002;81:1–4. 10.1016/s0378-8741(02)00097-1 12065163

[16] Comino C (2007). Isolation and functional characterization of a cDNA coding a hydroxycinnamoyltransferase involved in phenylpropanoid biosynthesis in Cynara cardunculus L. BMC Plant Biol.

[17] Comino C (2009). The isolation and mapping of a novel hydroxycinnamoyltransferase in the globe artichoke chlorogenic acid pathway. BMC Plant Biol.

[18] Curt MD, Sánchez G, Fernández J (2002). The potential of Cynara cardunculus L. for seed oil production in a perennial cultivation system. Biomass Bioenergy.

[19] Deidda M (1967). Contributo al miglioramento genetico del carciofo. Atti 1° Congr. Int. di Studi sul Carciofo, Bari.

[20] Dereeper A (2011). SNiPlay : a web-based tool for detection, management and analysis of SNPs.Application to grapevine diversity projects. BMC Bioinformatics.

[21] Encinar J (2002). Biodiesel fuels from vegetable oils: transesterification of Cynara cardunculus L. oils with ethanol. Energ Fuel.

[22] Encinar JM, González JF, González J (2000). Fixed-bed pyrolysis of Cynara cardunculus L. product yields and compositions. Fuel Process Technol.

[23] Falleh H (2008). Phenolic composition of Cynara cardunculus L. organs, and their biological activities. Comptes Rendus Biologies.

[24] Fernández J (1996). Aprovechamiento del cardo (Cynara cardunculus L.) para la producción de biomasa lignocelulósica, aceite y forraje verde. *Itea*. Producción Vegetal.

[25] Fernández J, Curt MD, Aguado PL (2006). Industrial applications of Cynara cardunculus L. for energy and other uses. Ind Crop Prod.

[26] Foury C (1969). Étude de la biologie florale de l’artichaut (Cynara scolymus L.). Application a la sélection 2° partie. Étude des descendances obtenues en fécondation contrôlée. Ann Amélior Plantes.

[27] Fratianni F (2007). Polyphenolic composition in different parts of some cultivars of globe artichoke (Cynara cardunculus L. var. scolymus (L.) Fiori). Food Chem.

[28] Galeano CH (2012). Gene-based single nucleotide polymorphism markers for genetic and association mapping in common bean. BMC Genet.

[29] Ganopoulos I, Argiriou A, Tsaftaris A (2011). Microsatellite high resolution melting (SSR-HRM) analysis for authenticity testing of protected designation of origin (PDO) sweet cherry products. Food Control.

[30] Gebhardt R (1998). Inhibition of cholesterol biosynthesis in primary cultured rat hepatocytes by Artichoke (Cynara scolymus L.) extracts. J Pharmacol Exp Ther.

[31] Gherbin P, Monteleone M, Tarantino E. Five year evaluation on Cardoon (Cynara cardunculus L. var. altilis) biomass production in Mediterranean Environment. Ital J Agron. 2001;11–19. Available at: http://www.siagr.org/public/rivista/5_1-2_2.pdf.

[32] Goldberg T, Hamp T, Rost B (2012). LocTree2 predicts localization for all domains of life. Bioinformatics.

[33] Gominho J, Fernandez J, Pereira H (2001). Cynara cardunculus L. — a new fibre crop for pulp and paper production. Ind Crop Prod.

[34] Gramazio P (2014). Location of chlorogenic acid biosynthesis pathway and polyphenol oxidase genes in a new interspecific anchored linkage map of eggplant. BMC Plant Biol.

[35] Han Y, Khu D-M, Monteros MJ (2012). High-resolution melting analysis for SNP genotyping and mapping in tetraploid alfalfa (Medicago sativa L.). Mol Breed.

[36] Hoffmann L (2003). Purification, cloning, and properties of an acyltransferase controlling shikimate and quinate ester intermediates in phenylpropanoid metabolism. J Biol Chem.

[37] Ierna A, Mauromicale G (2010). Cynara cardunculus L. genotypes as a crop for energy purposes in a Mediterranean environment. Biomass Bioenergy.

[38] Jeong H-J (2011). A survey of natural and ethyl methane sulfonate-induced variations of eIF4E using high-resolution melting analysis in Capsicum. Mol Breed.

[39] Jeong H-J (2010). Identification of Capsicum species using SNP markers based on high resolution melting analysis. Genome/National Research Council Canada = Genome/Conseil national de recherches Canada.

[40] Käll L, Krogh A, Sonnhammer ELL. Advantages of combined transmembrane topology and signal peptide prediction-the Phobius web server. Nucleic Acids Res. 2007;35(Suppl2):429–32. 10.1093/nar/gkm256 PMC1933244 17483518

[41] Komorowska B (2014). Simultaneous detection of Cherry necrotic rusty mottle virus and Cherry green ring mottle virus using real-time PCR and high resolution melting analysis. Mol Cell Probes.

[42] Kukić J (2008). Antioxidant and antimicrobial activity of Cynara cardunculus extracts. Food Chem.

[43] Küskü-Kiraz Z (2010). Artichoke leaf extract reduces oxidative stress and lipoprotein dyshomeostasis in rats fed on high cholesterol diet. Phytother Res.

[44] Lallemand LA (2012). A structural basis for the biosynthesis of the major chlorogenic acids found in coffee. Plant Physiol.

[45] Lanteri S (2004). Amplified fragment length polymorphism for genetic diversity assessment in globe artichoke. TAG Theoretical and applied genetics Theoretische und angewandte Genetik.

[46] Lehmensiek A, Sutherland MW, Mcnamara RB (2008). The use of high resolution melting (HRM) to map single nucleotide polymorphism markers linked to a covered smut resistance gene in barley.

[47] Lipscomb LA (1998). Context-dependent protein stabilization by methionine-to-leucine substitution shown in T4 lysozyme. Protein Sci.

[48] Llorach R (2002). Artichoke (Cynara scolymus L.) byproducts as a potential source of health-promoting antioxidant phenolics. J Agric Food Chem.

[49] Lobo V (2010). Free radicals, antioxidants and functional foods: impact on human health. Pharmacogn Rev.

[50] López Anido F (1998). Estimation of genetic parameters for yield traits in globe artichoke (Cynara scolymus L.). Euphytica.

[51] Lotfy S, Fleuriet A, Macheix J-J (1992). Partial purification and characterization of hydroxycinnamoyl CoA: transferases from apple and date fruits. Phytochemistry.

[52] Maccarone E (1999). Possible alternative utilization of Cynara spp.: II. Chemical characterization of their grain oil. Ind Crop Prod.

[53] Mackay JF, Wright CD, Bonfiglioli RG (2008). A new approach to varietal identification in plants by microsatellite high resolution melting analysis: application to the verification of grapevine and olive cultivars. Plant Methods.

[54] de Medeiros JM (2000). Antithrombin activity of medicinal plants of the Azores. J Ethnopharmacol.

[55] Menin B (2012). Genetic mapping and characterization of the globe artichoke (+)-germacrene A synthase gene, encoding the first dedicated enzyme for biosynthesis of the bitter sesquiterpene lactone cynaropicrin. Plant Sci.

[56] Mhlongo MI (2014). Metabolomic fingerprinting of primed tobacco cells provide the first evidence for the biological origin of cis-chlorogenic acid. Biotechnol Lett.

[57] Miccadei S (2008). Antioxidative and apoptotic properties of polyphenolic extracts from edible part of artichoke (Cynara scolymus L.) on cultured rat hepatocytes and on human hepatoma cells. Nutr Cancer.

[58] Mileo AM (2012). Artichoke polyphenols induce apoptosis and decrease the invasive potential of the human breast cancer cell line MDA-MB231. J Cell Physiol.

[59] Miller T (1975). New artichoke clones. New Zealand J Agr.

[60] Moglia A (2014). Dual catalytic activity of hydroxycinnamoyl-CoA quinate transferase from tomato allows it to moonlight in the synthesis of both mono- and dicaffeoylquinic acids. Plant Physiol.

[61] Moglia A (2009). Isolation and mapping of a C3′H gene (CYP98A49) from globe artichoke, and its expression upon UV-C stress. Plant Cell Rep.

[62] Moglia A (2008). Stress-induced biosynthesis of dicaffeoylquinic acids in globe artichoke. J Agric Food Chem.

[63] Mondini L (2012). Identification of SNP mutations in DREB1, HKT1, and WRKY1 genes involved in drought and salt stress tolerance in durum wheat (Triticum turgidum L. var durum). OMICS.

[64] Muleo R (2009). Mutation scanning and genotyping by high-resolution DNA melting analysis in olive germplasm. Genome/National Research Council Canada = Genome/Conseil national de recherches Canada.

[65] Nadova S (2008). Growth inhibitory effect of ethyl acetate-soluble fraction of Cynara cardunculus L. in leukemia cells involves cell cycle arrest, cytochrome c release and activation of caspases. Phytother Res.

[66] Niggeweg R, Michael AJ, Martin C (2004). Engineering plants with increased levels of the antioxidant chlorogenic acid. Nat Biotechnol.

[67] Pandino G (2015). Leaf polyphenol profile and SSR-based fingerprinting of new segregant Cynara cardunculus genotypes. Front Plant Sci.

[68] Petersen TN (2011). SignalP 4.0: discriminating signal peptides from transmembrane regions. Nat Meth.

[69] Piscioneri I (2000). Promising industrial energy crop, Cynara cardunculus: a potential source for biomass production and alternative energy. Energy Convers Manag.

[70] Raccuia SA, Melilli MG (2007). Biomass and grain oil yields in Cynara cardunculus L. genotypes grown in a Mediterranean environment. Field Crop Res.

[71] Radivojac P (2010). Identification, analysis, and prediction of protein ubiquitination sites. Proteins.

[72] Ramos PAB (2014). Phenolic composition and antioxidant activity of different morphological parts of Cynara cardunculus L. var. altilis (DC) Patrícia A. B. Ramos. Ind Crop Prod.

[73] Ramos PAB (2013). Lipophilic extracts of Cynara cardunculus L. var. altilis (DC): a source of valuable bioactive terpenic compounds. J Agric Food Chem.

[74] Rottenberg A, Zohary D, Nevo E (1996). Isozyme relationships between cultivated artichoke and the wild relatives. Genet Resour Crop Evol.

[75] Sánchez-Moreno C, Larrauri JA, Saura-Calixto F (1998). A procedure to measure the antiradical efficiency of polyphenols. J Sci Food Agric.

[76] Scherer R, Godoy HT (2009). Antioxidant activity index (AAI) by the 2,2-diphenyl-1-picrylhydrazyl method. Food Chem.

[77] Sonnante G (2011). Genetic map of artichoke × wild cardoon: toward a consensus map for Cynara cardunculus. Theor Appl Genet.

[78] Sonnante G (2010). Novel hydroxycinnamoyl-coenzyme A quinate transferase genes from artichoke are involved in the synthesis of chlorogenic acid. Plant Physiol.

[79] Sonnante G, Pignone D, Hammer K (2007). The domestication of artichoke and cardoon: from Roman times to the genomic age. Ann Bot.

[80] Sprung R (2008). Identification and validation of eukaryotic aspartate and glutamate methylation in proteins. J Proteome Res.

[81] St-Pierre B (1998). The terminal O-acetyltransferase involved in vindoline biosynthesis defines a new class of proteins responsible for coenzyme A-dependent acyl transfer. Plant J.

[82] Suzuki H, Nakayama T, Nishino T (2003). Proposed mechanism and functional amino acid residues of Malonyl-CoA:Anthocyanin 5-O-Glucoside-6‘“-O-Malonyltransferase from flowers of Salvia splendens, a member of the versatile plant Acyltransferase family. Biochemistry.

[83] Tan YY (2013). Functional molecular markers and high-resolution melting curve analysis of low phytic acid mutations for marker-assisted selection in rice. Mol Breed.

[84] Tesi R (1976). Primi risultati del miglioramento genetico nelle varieta toscane de Cynara cardunculus v. scolymus. Atti 2° Congr. Int. di Studi sul Carciofo, Bari.

[85] Ulbrich B, Zenk MH (1979). Partial purification and properties of hydroxycinnamoyl-CoA: quinate hydroxycinnamoyl transferase from higher plants. Phytochemistry.

[86] Velez Z, et al. Biological characterization of Cynara cardunculus L. methanolic extracts: antioxidant, anti-proliferative. Anti-migratory and Anti-angiogenic Activities Agriculture. 2012;2(4):472–92. Available at: http://www.mdpi.com/2077-0472/2/4/472/. Accessed 13 Aug 2014].

[87] Wang C (2013). Loop nucleotide polymorphism in a putative miRNA precursor associated with seed length in rice (Oryza sativa L.).

[88] Wang M (2003). Analysis of antioxidative phenolic compounds in artichoke (Cynara scolymus L.). J Agric Food Chem.

[89] Wittwer CT, et al. High-resolution genotyping by amplicon melting analysis using LCGreen. 2003;860:853–60. 10.1373/49.6.853 12765979

[90] Wu S-B (2008). High resolution melting analysis of almond SNPs derived from ESTs. TAG Theoretical and applied genetics Theoretische und angewandte Genetik.

[91] Xia D-Z (2011). Antioxidant and antibacterial activity of six edible wild plants (Sonchus spp.) in China. Nat Prod Res.

[92] Zhao J (2007). Nutraceuticals, nutritional therapy, phytonutrients, and phytotherapy for improvement of human health: a perspective on plant biotechnology application. Recent Pat Biotechnol.

[93] Zhu X, Zhang H, Lo R (2004). Phenolic compounds from the leaf extract of artichoke (Cynara scolymus L.) and their antimicrobial activities. J Agric Food Chem.

[94] Zhu XF, Zhang HX, Lo R. Antifungal activity of Cynara scolymus L. extracts. Fitoterapia. 2005;76(1):108–11. Available at: http://www.sciencedirect.com/science/article/pii/S0367326X04002461. 10.1016/j.fitote.2004.10.016 15664472

