# Supplementary figures and images for: Impact of novel SNPs identified in Cynara cardunculus genes on functionality of proteins regulating phenylpropanoid pathway and their association with biological activities
Source: BMC Genomics. 2017 Feb 17;18:183. doi: 10.1186/s12864-017-3534-8 (PMC5314637; doi:10.1186/s12864-017-3534-8)

**C3'H**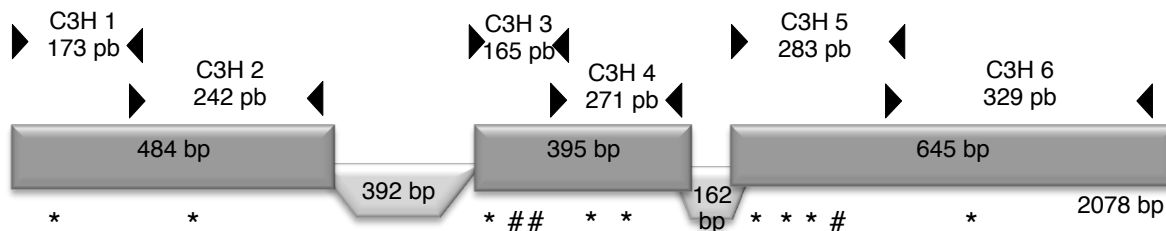**HQT**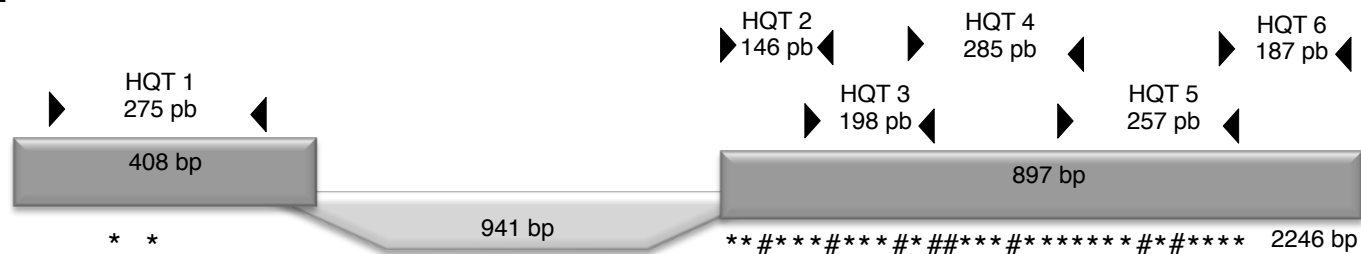

Supplement: Additional file 2: — C3′H and HQT gene structures and primer pairs (triangle arrows) designed for HRM analysis. (*) SNPs position; (#) a.a. alteration. The introns are indicated in grey. (PDF 57 kb) [file 12864_2017_3534_MOESM2_ESM.pdf]
